# *Dirofilaria immitis* Microfilariae and Third-Stage Larvae Induce Canine NETosis Resulting in Different Types of Neutrophil Extracellular Traps

**DOI:** 10.3389/fimmu.2018.00968

**Published:** 2018-05-08

**Authors:** Tamara Muñoz-Caro, Iván Conejeros, Ershun Zhou, Anton Pikhovych, Ulrich Gärtner, Carlos Hermosilla, Daniel Kulke, Anja Taubert

**Affiliations:** ^1^Institute of Parasitology, Biomedical Research Center Seltersberg, Justus Liebig University Giessen, Giessen, Germany; ^2^Clinical Development Animal Health, Animal Center, Bayer Animal Health GmbH, Leverkusen, Germany; ^3^Institute of Anatomy and Cell Biology, Justus Liebig University Giessen, Giessen, Germany; ^4^Drug Discovery Animal Health, Parasiticides, Filaricides Research, Bayer Animal Health GmbH, Leverkusen, Germany

**Keywords:** *Dirofilaria immitis*, neutrophil extracellular traps, innate immunity, NETosis, canine polymorphonuclear neutrophils

## Abstract

Heartworm disease is a zoonotic vector-borne disease caused by *Dirofilaria immitis* mainly affecting canids. Infectious third-stage larvae (L3) are transmitted to the definitive hosts *via* culicid mosquitoes; adult nematodes reside in the pulmonary arteries and in the right heart releasing unsheathed first-stage larvae (microfilariae) into the bloodstream leading to chronic and sometimes fatal disease. So far, early innate immune reactions triggered by these different *D. immitis* stages in the canine host have scarcely been investigated. Therefore, *D. immitis* microfilariae and L3 were analyzed for their capacity to induce neutrophil extracellular traps (NETs) in canine polymorphonuclear neutrophils (PMN). Overall, scanning electron microscopy analysis revealed both larval stages as strong inducers of canine NETosis. Co-localization of PMN-derived extracellular DNA with granulocytic histones, neutrophil elastase, or myeloperoxidase in parasite-entrapping structures confirmed the classical characteristics of NETosis. Quantitative analyses showed that both larval stages triggered canine NETs in a time-dependent but dose-independent manner. Moreover, parasite-induced NET formation was not influenced by the parasites viability since heat-inactivated microfilariae and L3 also induced NETs. In addition, parasite/PMN confrontation promoted significant entrapment but not killing of microfilariae and L3. Both, NETosis and larval entrapment was significantly reversed *via* DNase I treatments while treatments with the NADPH oxidase inhibitor diphenyleneiodonium failed to significantly influence these reactions. Interestingly, different types of NETs were induced by microfilariae and L3 since microfilarial stages merely induced spread and diffuse NETs while the larger L3 additionally triggered aggregated NET formation.

## Introduction

Cardiopulmonary dirofilariosis or heartworm disease is a zoonotic vector-borne disease caused by the parasitic nematode *Dirofilaria immitis* affecting mainly dogs in warm-temperate regions, particularly in the southern United States, Central and South America, East Asia, and Mediterranean regions of Europe (1). Parasites are transmitted as third-stage larvae (L3) by infected culicid female mosquitoes of the genera *Culex, Aedes*, and *Anopheles* during the blood meal. Adult *D. immitis* nematodes reside in the pulmonary arteries and the right heart of definitive hosts where mature viviparous females release unsheathed first-stage larvae (microfilariae) into the bloodstream. Initial inflammatory responses occur in the walls of the pulmonary vasculature and are critical for pathogenesis and further development of the disease (2). This chronic and progressive infection may have a fatal outcome for definitive hosts and clinical cardiopulmonary disease has been reported in several carnivores, including domestic and wild canids [e.g., foxes, wolves (3)] and felids. Feline infections are increasingly reported from areas endemic for canine dirofilariosis, but generally show a low parasite burden and asymptomatic infections. In some cases, cats may present severe disease or even sudden death even in the presence of a small number of adult worms (4–8). Currently, cardiopulmonary dirofilariosis is considered as an emerging disease in Europe, most probably due to climate change favoring intermediate host expansion, inadequate management of pets, human intervention in the environment, and the existence of wild reservoirs (9–11).

So far, little data are available on innate immune reactions against *D. immitis*. The fact, that polymorphonuclear neutrophils (PMN) are critically involved in the killing and clearance of microfilariae *via* antibody-dependent cell-mediated cytotoxicity *in vitro* and from dogs with occult *D. immitis* infections (12, 13), indicates an important role of this innate immune cell type. PMN showed enhanced oxidative burst and degranulation activities in response to opsonized microfilariae (14) and adhered to *D. immitis* microfilariae in the absence of immune serum and presence of ivermectin (15, 16). In addition, PMN infiltration was observed in various organs and tissues, including kidney and arterial walls from *D. immitis* infected canids (17, 18). Accordingly, several parasite-derived chemotactic factors were demonstrated to attract PMN (19). Interestingly, endosymbiotic bacteria of the genus *Wolbachia* residing within *D. immitis* adults (20) were shown to activate canine PMN leading to enhanced production of IL-8 (21). Concerning other filarial parasites, PMN were shown to promote the killing of *Onchocerca volvulus* microfilariae (unsheathed) and L3 *in vitro* (22, 23) and played a major role in the early control of *Litomosoides sigmodontis* L3 stages in the skin leading to enhanced oxidative burst and degranulation (24).

The effector mechanism of NETosis is performed by PMN and other immune cells and results in the cellular release of granule proteins and chromatin upon activation, forming sticky extracellular fibers capable of binding and killing Gram-positive and -negative bacteria, fungi, virus, and protozoan/metazoan parasites (25–28). So far, the capacity of extracellular trap formation has been attributed to PMN (25), mast cells (29), macrophages (30), eosinophils (31), and monocytes (32–34). NETosis is most of the time mediated by NADPH oxidase (NOX)-dependent ROS production (35, 36) and efficient neutrophil extracellular trap (NET) extrusion is a cell death-dependent process in the majority of cases. However, non-lethal NET release was also reported by Yousefi et al. (31, 37) who demonstrated that eosinophils and certain PMN subpopulations release NETs of mitochondrial origin without dying. In addition, recent studies verified that PMN were still viable and retained their capability to engulf bacteria *via* phagocytosis even after performing NETosis (38).

So far, most studies on parasite-triggered NETosis focused on protozoan parasites (38–45). By contrast, little data exist on metazoan-triggered NET formation (46–50). In the case of filariae, the reports on NETosis come exclusively from *Brugia malayi* and *L. sigmodontis*, in which microfilariae/L3 were recently demonstrated as potent triggers of NETs leading to parasite entrapment but not killing *in vitro* (24, 50). It is worth noting that adult *D. immitis* worms survive for an average of 5 years within the definitive host, releasing microfilariae into the bloodstream (51). However, the immune mechanisms that drive host tolerance and the modulation of host innate immune responses driven by blood circulating microfilariae are not yet fully understood. In addition, no data are available on the capacity of *D. immitis* stages as NET inducers in the canine host.

In this study, we show that microfilariae and L3 from the *D. immitis* Missouri strain, USA, significantly induce NET formation and trigger different forms of NETs in a stage-specific manner promoting additional and novel innate immune reactions of canine PMN against this parasite.

## Materials and Methods

### Parasites

For all here described experiments, the *D. immitis* Missouri strain, USA, was used. This strain originated from a dog sheltered in an animal pound in Missouri in the year 2000. Beginning in 2005, the strain was maintained and passaged in beagle dogs at the University of Georgia (Athens, GA, USA) (52). From 2012 onward, the strain was maintained at the laboratories of Bayer Animal Health, Monheim, Germany. Here, all animal procedures were approved by the local animal care and use committee and by governmental authorities (LANUV #200/A176 and #200/A154). For this study, microfilaremic blood was sampled from four dogs (Beagle, Marshall BioResources, two males and two females) by cephalic vein puncture. Microfilariae were purified from the blood samples according to the protocol provided by the NIH/NIAID Filariasis Research Reagent Resource Center (FR3; College of Veterinary Medicine, University of Georgia, Athens, GA, USA) (http://www.filariasiscenter.org/protocols/Protocols/purification-of-microfilariae-by-filtration). L3 were obtained by feeding microfilaremic canine blood to *Aedes aegypti* (black-eyed Liverpool strain). Fifteen days after feeding, L3 were isolated from infected *A. aegypti* according to the protocol by Evans et al. (52).

### Canine PMN Isolation

In total, blood sampling for PMN isolation was performed on six adult dogs (Beagle, Marshall BioResources) by jugular vein puncture. In each experimental setting, at least three different blood donors were included. Heparinized blood was diluted in an equal amount of PBS containing 0.02% EDTA, layered on Biocoll^®^ Separating Solution (Biochrom AG) and centrifuged (800 × *g*, 45 min). The pellet was gently resuspended and shaken in 25 ml distilled water for 20 s to lyse erythrocytes. Then, osmolarity was adjusted by adding 5 ml of 10× Hanks Salt Solution (HBSS, Biochrom AG). Afterward, PMN were washed twice (400 × *g*, 10 min), resuspended in RPMI medium, counted in a Neubauer hemocytometer chamber, and incubated at 37°C and 5% CO_2_ for at least 30 min before experimental use.

### Scanning Electron Microscopy (SEM)

Canine PMN (*n* = 3.5 × 10^5^) were cocultured with vital *D. immitis* L3 (10 larvae/sample) or microfilariae (20 larvae/sample) on poly-l-lysine (Sigma-Aldrich) pre-coated coverslips (60 min, 37°C). After incubation, the samples were fixed in 2.5% glutaraldehyde [60 min, room temperature (RT), Merck], post-fixed in 1% osmium tetroxide (Merck), washed in distilled water, dehydrated, critical point dried by CO_2_ treatment, and spayed with gold. Thereafter, samples were examined with a Philips XL30 scanning electron microscope at the Institute of Anatomy and Cell Biology, Justus Liebig University Giessen, Germany.

### NET Visualization by Immunofluorescence Analysis

Canine PMN (*n* = 3; 2 × 10^5^/300 μl) were seeded on 15 mm poly-l-lysine-treated glass coverslips placed in 12-well plates in RPMI 1640 medium (without phenol red, supplemented with 1% penicillin/streptomycin, Sigma-Aldrich) and cocultured with *D. immitis* microfilariae (50 larvae/well; 37°C, 5% CO_2_, 60 min). After the incubation period, the samples were fixed in 4% paraformaldehyde (Merck) and kept at 4°C until further use. Canine NET structures were visualized by staining extracellular DNA with Sytox Orange^®^ (1:1,000, 15 min, Invitrogen) according to Martinelli et al. (53). For the detection of histones, myeloperoxidase (MPO) and neutrophil elastase (NE) within NET structures the following antibodies were used: anti-histone (H1, H2A/H2B, H3, H4) (clone H11-4, 1:1,000; Merck Millipore), anti-MPO (orb11073, 1:1,000, Byorbit), and anti-NE (AB68672, 1:1,000, Abcam). Briefly, the samples were washed thrice in PBS, blocked with 2% bovine serum albumin (Sigma-Aldrich; 30 min, RT) and incubated in antibody solutions (60 min, RT). Thereafter, the samples were washed thrice with PBS and incubated in secondary antibody solutions (Alexa Fluor 488 goat anti-mouse IgG or Alexa Fluor 488 goat anti-rabbit IgG, both Life Technologies, 60 min, 1:1,000, RT). Finally, the samples were washed thrice in PBS and mounted in anti-fading buffer (ProLong Gold Antifade Mountant^®^; Thermo Fisher Scientific). Visualization was achieved using an inverted Olympus IX81 fluorescence microscope equipped with a digital camera (Olympus).

### Quantification of Extracellular DNA Release

Using 96-well flat bottom plates (Nunc), canine PMN (*n* = 3, 2 × 10^5^ in duplicates) were cocultured with *D. immitis* microfilariae (100 and 300 larvae per sample) or L3 (10 and 20 larvae per sample) in RPMI 1640 medium (1% penicillin/streptomycin, without phenol red) in a final volume of 200 µl. In parallel, PMN were pretreated with the NADPH inhibitor diphenyleneiodonium (DPI, 10 µM 30 min; Sigma-Aldrich) before exposure to microfilariae. To resolve NET formation, treatment with DNase I (90 U/sample, Roche Diagnostics) was used. For heat-induced killing (HI), microfilariae or L3 were incubated for 60 min at 60°C. Following coculture, 3 µM Sytox Orange^®^ Nucleic Acid Stain (Life Technologies, final concentration) was added to each well, and measurements were performed every 30 min for up to 8 h. Sytox Orange-derived fluorescence intensities were estimated by spectrofluorometric analyses at an excitation wavelength of 547 nm and emission wavelength 570 nm using an automated plate monochrome reader (Varioskan Flash^®^; Thermo Scientific). For negative controls, PMN in plain medium were used. Stimulation of PMN with zymosan (1 mg/ml; Invitrogen) served as positive control.

### Attachment and Entrapment Assay

Canine PMN (*n* = 3, 2 × 10^5^ in duplicates) were seeded on poly-l-lysine pre-coated coverslips and exposed to *D. immitis* microfilariae (50 larvae/sample) or L3 (10 larvae/sample) in a final volume of 200 µl for 60 and 180 min (37°C, 5% CO_2_). In parallel settings, PMN were exposed to heat-killed (HI) microfilariae or pretreated with 10 µM DPI (Sigma-Aldrich) for 30 min before exposure to microfilariae. Treatments of PMN with 90U DNase I (Fisher Scientific) at the moment of parasite exposure were used to dissolve NETs. Following incubation, all samples were fixed (4% paraformaldehyde), and PMN-entrapped larvae were scored microscopically by using an inverted DMIRB^®^ phase-contrast microscope (Leica). Larvae were considered as entrapped when PMN, and/or PMN-derived NETs were attached to the larvae. The data were expressed as percentage of entrapped larvae relative to the total amount of larvae.

### Assessment of Different Types of NETs

For the quantification of different types of NETs, canine PMN (*n* = 3, 2 × 10^5^ in duplicates) were seeded on poly-l-lysine pre-coated coverslips and exposed to *D. immitis* microfilariae (50 larvae/sample) and L3 (10 larvae/sample) for 60 and 180 min (37°C, 5% CO_2_). Thereafter, the samples were fixed (4% paraformaldehyde) for further analysis. NET structures were visualized by staining extracellular DNA with Sytox Orange^®^ (1:1,000, 15 min; Invitrogen) and were reacted with an anti-global histone antibody (clone H11-4 monoclonal antibody, 1:1,000; Merck Millipore) as previously described. Thereafter, four randomly selected images from each experimental condition were analyzed microscopically based on typical morphological characteristics according to Muñoz-Caro et al. (48) and Lange et al. (49): (i) “diffuse” NETs (*diff*NETs), which are composed of a complex of extracellular decondensed chromatin mesh being decorated with antimicrobial proteins with globular and compact form, defined by a size of 15–20 μm diameter, (ii) “spread” NETs (*spr*NETs) consisting of smooth and elongated web-like structures of decondensed chromatin and antimicrobial proteins and being composed exclusively of thin fibers with a diameter of 15–17 μm, and (iii) “aggregated” NETs (*agg*NETs), being defined as clusters of NET-like structures with a “ball of yarn” morphology and a size of more than 20 µm in diameter. Within each sample, all structures with the described characteristics were counted.

### Analysis of NET-Derived Larval Killing

Canine PMN (*n* = 3, 2 × 10^5^) were seeded in 12-well culture well plates and exposed to *D. immitis* microfilariae (50 larvae/sample) or L3 (10 larvae/sample) for 24, 36, 48, and 72 h (37°C, 5% CO_2_). Larval survival was determined microscopically based on the presence or absence of larval motility and monitored *via* video recording. For reference samples, the equal number of non-exposed larvae was used.

### Statistical Analysis

Given that the data were normally distributed, coculture/stimulation conditions were compared by one- or two-factorial analyses of variance (ANOVA) with repeated measures. Differences were regarded as significant at a level of *p* ≤ 0.05 and analyzed by GraphPad Prism^®^ programme. To analyze time dependency, a 2-way repeated measures ANOVA, followed by a Tukey’s multiple comparisons test were performed. The repeated measure category was time, and the α value to consider *p* significant between the experimental conditions was 0.05. Data were plotted as mean (SD).

## Results

### *D. immitis* Microfilaria and L3 Induce Canine NETosis in a Time-Dependent Manner

**Figure 1:**
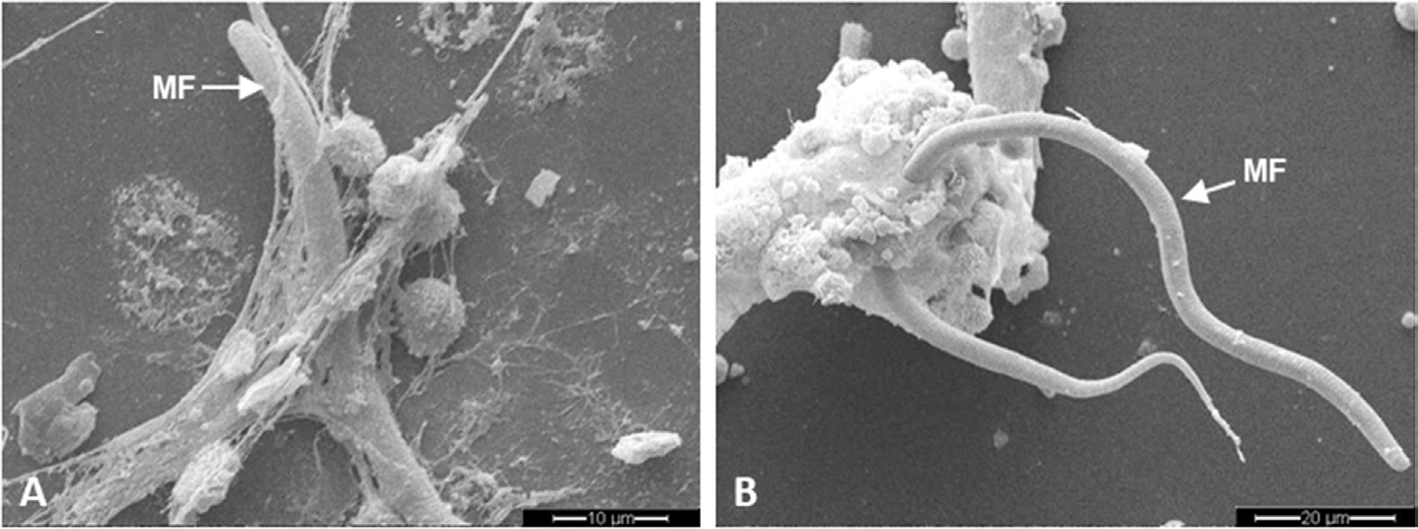
*Dirofilaria immitis*-induced neutrophil extracellular trap (NET) formation analyzed *via* scanning electron microscopy analysis. Coculture of canine polymorphonuclear neutrophils with *D. immitis* microfilariae (MF). **(A)** Fine spread NETs entrapping microfilariae. **(B)** Meshwork of aggregated NETs (*agg*NETs) entrapping microfilariae.

Scanning electron microscopy analyses revealed that exposure of canine PMN to *D. immitis* microfilariae resulted in rapid and strong formation of NETs since fine and filamentous but also robust DNA-derived structures contacted and ensnared these unsheathed microfilarial stages (Figure 1).

**Figure 2:**
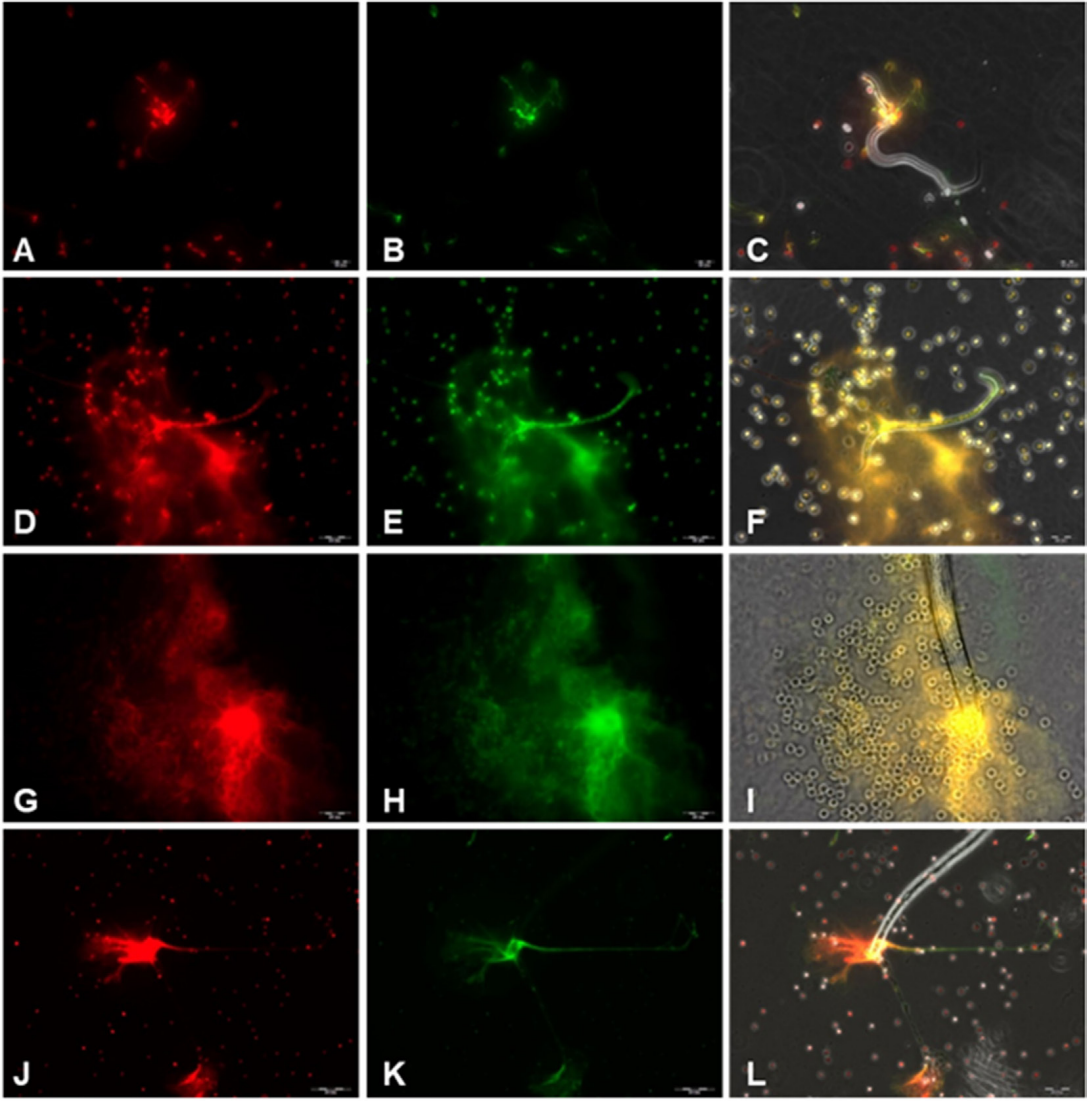
Immunofluorescence analyses on *Dirofilaria immitis* microfilariae-induced neutrophil extracellular trap (NET) formation. Co-localization experiments on extracellular DNA and histones, myeloperoxidase (MPO), and neutrophil elastase (NE) in *D*. *immitis* microfilariae-induced NET structures using DNA-marker Sytox Orange and anti-histone H1, H2A/H2B, H3, H4, anti-MPO, and anti-NE antibodies. **(A,D,G,J)** Extracellular DNA stained with Sytox Orange (red). **(B)** Anti-histone (green). **(E,H)** Anti-NE (green). **(K)** Anti-MPO (green). **(C,F,I,L)** Merges.

To confirm the classical NETs components in parasite-triggered structures, fluorescence-based analyses were performed. Thus, Sytox Orange^®^ staining (Figures 2A,D,G,J) proved the DNA nature of extracellular NET-like structures being formed by canine PMN upon exposure to *D. immitis* parasites. In addition, co-localization studies revealed the simultaneous presence of MPO, NE and histones (H1, H2A/H2B, H3, and H4) (Figures 2B,E,H,K) in DNA-positive parasite-induced NET structures (Figures 2C,F,I,L). It is noteworthy that a fast induction of NETs was observed *via* both stages, microfilariae and L3, since respective reactions were visible already 10 min after coculture (Image S1 (A and B) in Supplementary Material).

**Figure 3:**
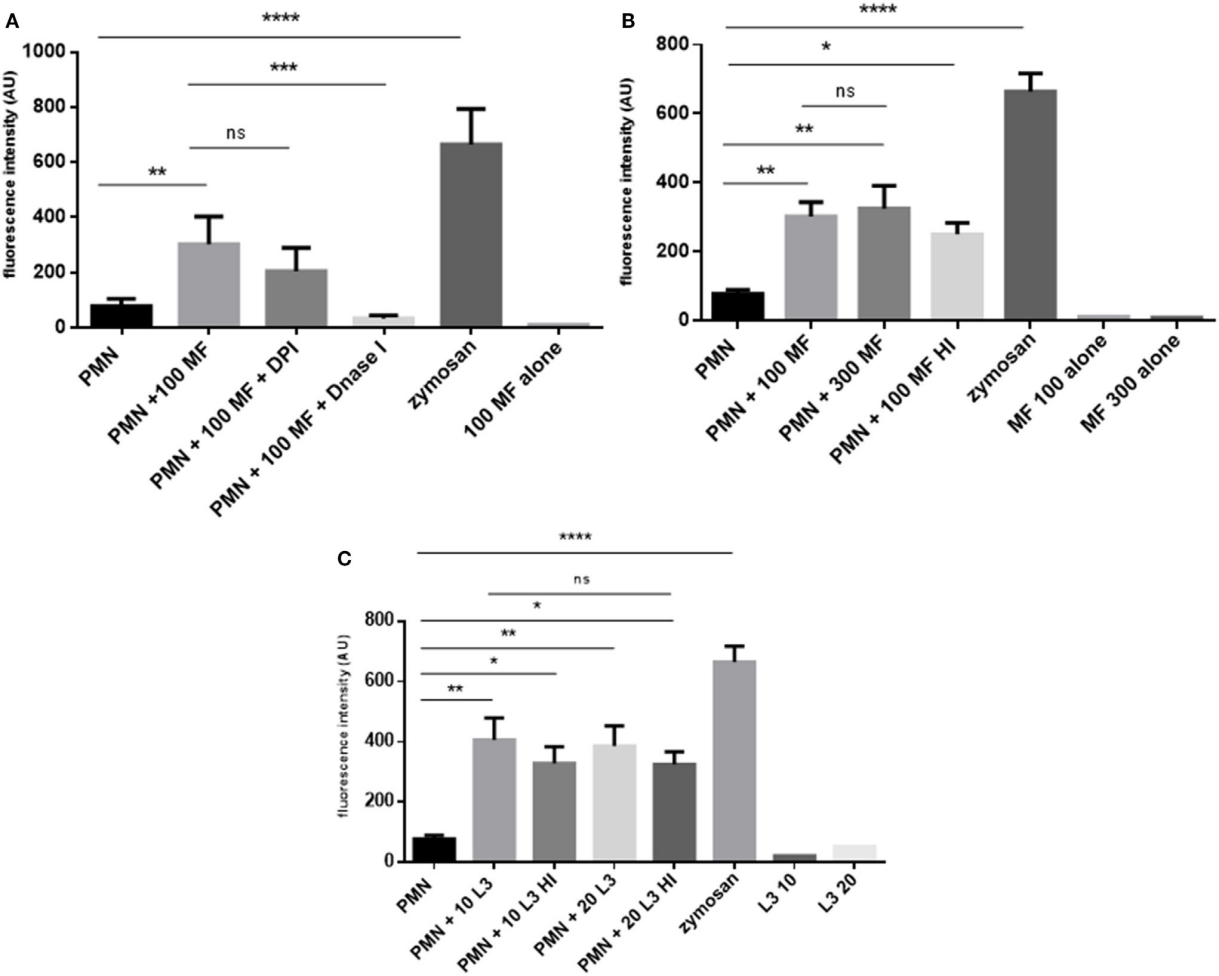
*Dirofilaria immitis*-induced dose- and viability- and diphenyleneiodonium (DPI)-independent neutrophil extracellular trap (NET) formation. **(A)** *D. immitis* microfilariae (MF; 100) were cocultured with canine polymorphonuclear neutrophils (PMN) for 180 min. For NADPH oxidase inhibition, DPI pre-treatment was used. To resolve NET formation, DNase I was added to coculture. **(B)** Canine PMN were cocultured with vital or heat-inactivated microfilariae (MF-HI). **(C)** Vital and heat-inactivated *D. immitis* L3 were exposed to canine PMN. Sytox Orange-derived fluorescence intensities were analyzed by spectrofluorometric analysis at an excitation wavelength of 547 nm and emission wavelength 570 nm using an automated plate monochrome reader. As negative control, PMN in plain medium were used. PMN stimulated with zymosan served as positive control.

**Figure 4:**
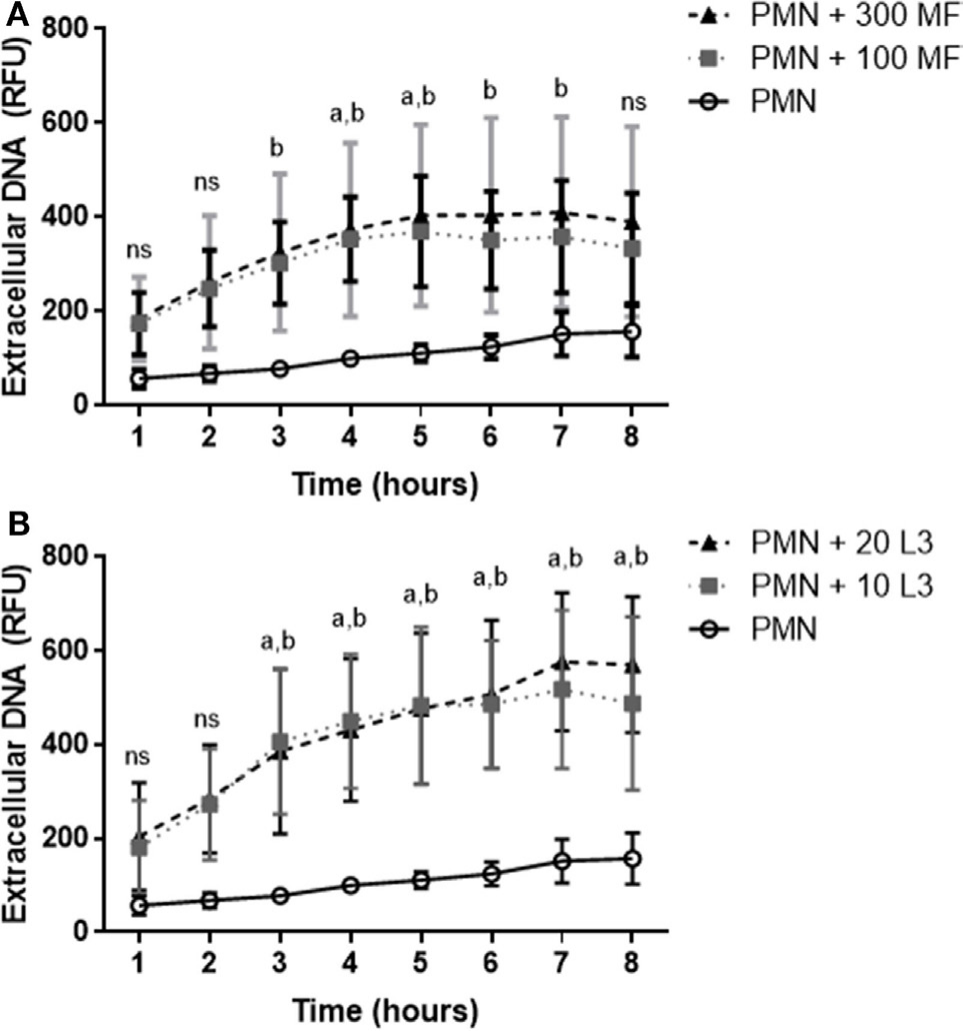
Time dependency of *Dirofilaria immitis*-induced neutrophil extracellular traps. Canine polymorphonuclear neutrophils (PMN) were cocultured with microfilariae **(A)** or L3 **(B)** for 8 h. Sytox Orange-positive signals were analyzed every hour by spectrofluorometric analysis using an automated plate monochrome reader. **(A)** a: significant difference between PMN and 100 microfilariae; b: significant difference between PMN and 300 microfilariae. **(B)** a: significant difference between PMN and 10 L3; b: significant difference PMN and 20 L3. Abbreviation: ns, not significant.

Quantitative analyses confirmed that both, microfilariae and L3 significantly induced NET formation in canine PMN (*p* ≤ 0.01, Figures 3A–C). As expected, these reactions were significantly and almost entirely resolved by DNase I treatments (*p* ≤ 0.001, Figure 3A). To elucidate the role of NOX in *D. immitis*-triggered NETosis, functional inhibition experiments were performed using DPI as blocker of NOX. Here, DPI treatments failed to significantly reduce microfilariae-induced NET formation (Figure 3A), suggesting that this was a NOX-independent process. Furthermore, microfilariae- and L3-induced NET formation proved as independent on the parasite vitality since heat-killed microfilariae (MF-HI) and L3 (L3-HI) induced comparable levels of NETs when compared with non-treated parasite stages (Figures 3B,C). In addition, microfilariae- and L3-triggered NET formation revealed as dose-independent since enhanced numbers of parasitic stages did not significantly alter the level of NET induction (Figures 3B,C). Kinetic analysis on *D. immitis* microfilariae- and L3-induced NETosis showed a time-dependent pattern since NET formation was found increasingly enhanced (Figure 4A) in the case of 100 microfilariae (*p* ≤ 0,05; 4 h) and 300 microfilariae (*p* ≤ 0.05; 3 h) as well as 10 (*p* ≤ 0.05; 3 h) and 20 L3 (*p* ≤ 0.05; 3 h) (Figure 4B). Again, no dose-dependency was detected in these assays.

In all experiments, the stimulation with zymosan proved as reliable positive control (zymosan treatment vs. PMN in plain medium: *p* ≤ 0.0001). Microfilariae and L3 stages alone as well as PMN alone showed almost no or low reactions in this experimental setting, respectively.

### *D. immitis* Microfilariae and L3 Induce Different Types of NETs

**Figure 5:**
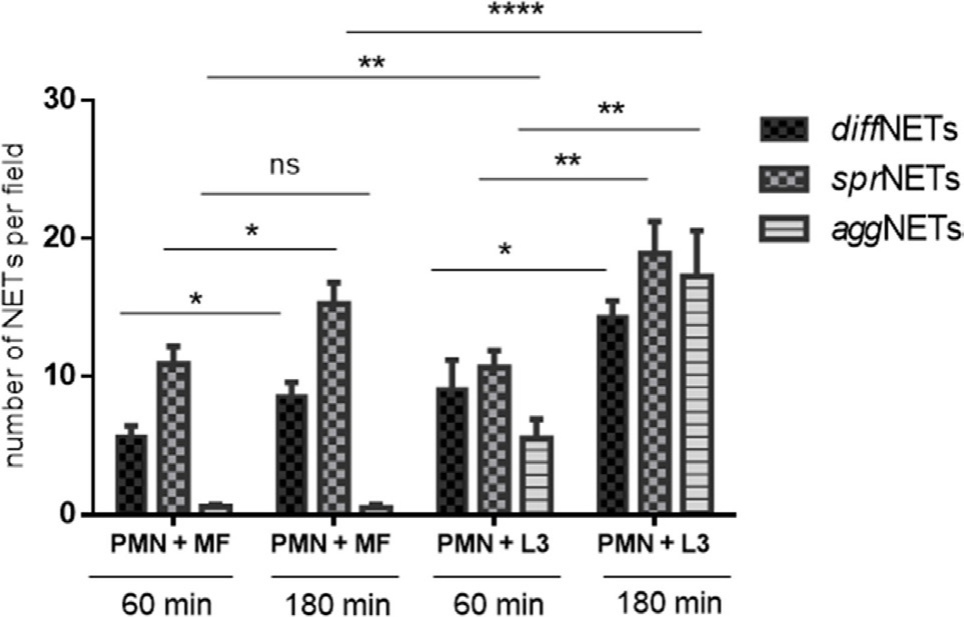
Quantification of different types of neutrophil extracellular traps (NETs) induced by *Dirofilaria immitis* stages. Canine polymorphonuclear neutrophils (PMN) (*n* = 3, 2 × 10^5^) we exposed to microfilariae (MF; 50 larvae) and L3 (10 larvae) on poly-l-lysine pre-coated coverslips for 1 and 3 h. After fixation, immunofluorescence analysis was performed using Sytox Orange as DNA-marker along with anti-histone antibodies. The types of NETs (*diff*NETs, *spr*NETs, and *agg*NETs) were identified microscopically and counted.

Overall, NETs may be displayed in different morphological forms, i.e., as diffuse NETs (*diff*NETs), aggregated NETs (*agg*NETs), and spread NETs (*spr*NETs). Thus, we here analyzed microscopically if microfilariae and L3 stages of *D. immitis* induced different types of NETs (as exemplary illustrations, please refer to Images S2 and S3 in Supplementary Material). In principle, all types of NETs were observed in PMN/microfilariae cocultures (Figure 5). Quantitative assessment of the different NET types revealed that *diff*NETs and *spr*NETs were induced by both parasitic stages, while *agg*NETs were mainly triggered by the larger larval stages (L3) (Figure 5). This stage-dependent difference was highly significant (*agg*NETs/microfilariae vs. *agg*NET/L3: *p* ≤ 0.01). Furthermore, microfilarial stages more frequently induced *spr*NETs than *diff*NETs (*p* ≤ 0.01) or *agg*NETs (*p* ≤ 0.001). For *spr*NET and *diff*NET formation, these reactions revealed as time-dependent and increased significantly with longer duration of coculture (*p* ≤ 0.05) (Figure 5). By contrast, the case of L3 all forms of NETs were induced at comparable levels but also showed a time-dependent pattern for all three types of NETs (*diff* NETs: *p* ≤ 0.05; *spr*NETs and *agg*NETs: *p* ≤ 0.01) (Figure 5).

### *D. immitis* Stages Are Entrapped *via* NETs in a Time-Dependent Manner

**Figure 6:**
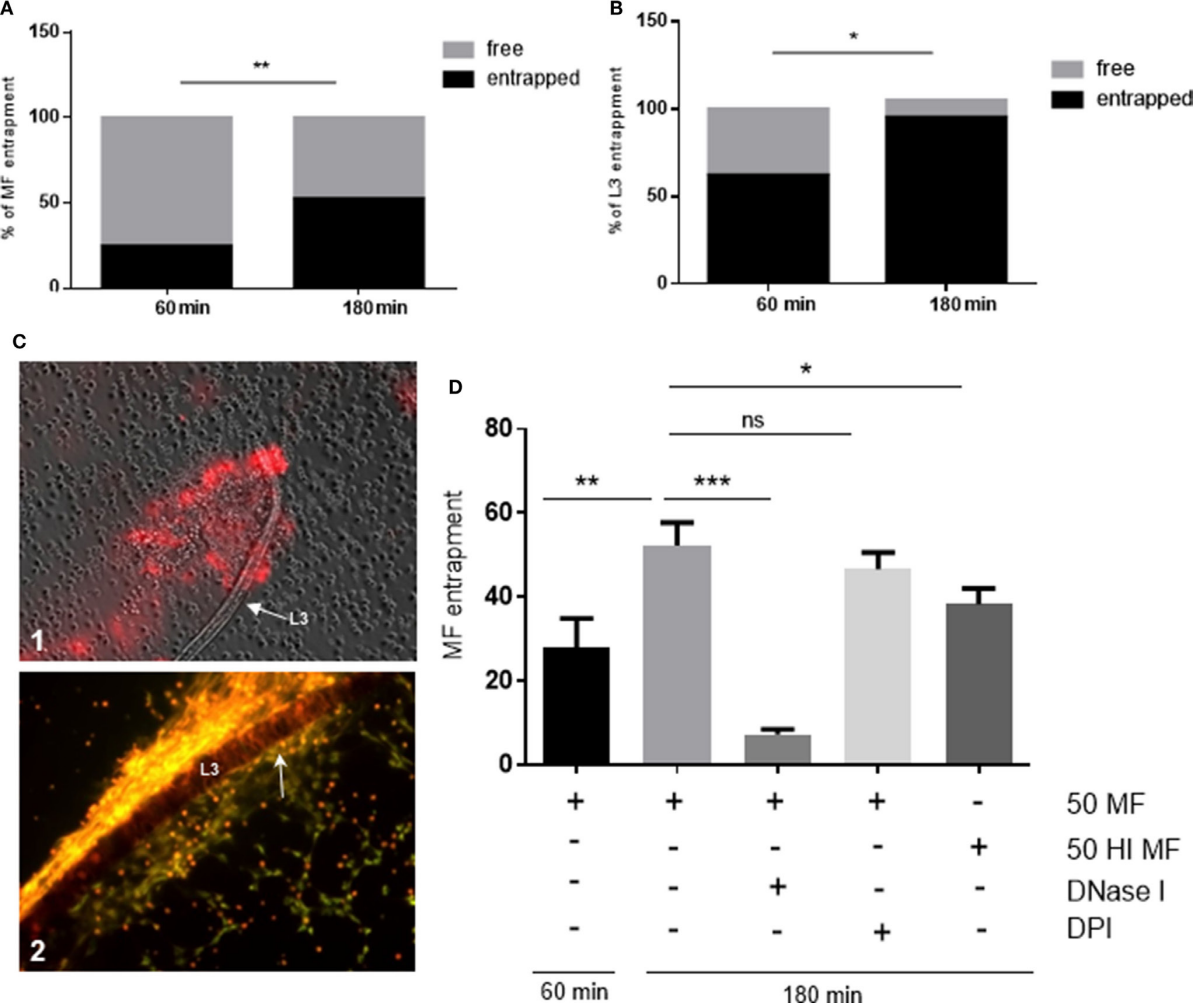
*Dirofilaria immitis-*induced parasite entrapment. Canine polymorphonuclear neutrophils (PMN) were exposed to vital *D. immitis* microfilariae **(A)** or L3 **(B,C)** for 60 and 180 min. **(D)** In parallel settings, the same number of PMN was incubated either with heat-inactivated (HI) microfilariae (HI MF) or pretreated with diphenyleneiodonium (DPI) before exposure to vital microfilariae. Furthermore, DNase I was added at the moment of exposure to vital microfilariae. Larvae were considered as entrapped when PMN and/or PMN-derived neutrophil extracellular traps (NETs) were in contact with larvae. The data are expressed as percentage of entrapped larvae relative to the total amount of larvae per condition. The formation of “clasp”-like NET structures sticking mainly to the anterior part of the larvae is displayed in white arrow: image [**(C)**, 2].

Since *D. immitis* microfilariae and L3 were proven as NET inducers in quantitative experiments, we here analyzed which proportion of these larval stages were physically contacted by PMN and entrapped by NET structures. Here, a time-dependent increase of parasite entrapment was observed for both stages. As such, a significant increase of parasite entrapment was observed in PMN/microfilariae cocultures at 60 and 180 min exposure (*p* ≤ 0.01, Figure 6A) leading to a 28 and 52% larval entrapment, respectively. Similar reactions were observed for L3 stages since 62 and 95% were found entrapped at 60 and 180 min of incubation (*p* ≤ 0.05, Figure 6B). When comparing the two larval stages, L3 were generally contacted and entrapped to a higher degree than microfilariae [28% (microfilariae) vs. 62% (L3) at 60 min, and 52% (microfilariae) vs. 95% (L3) at 180 min, both *p* ≤ 0.05, Figure 6B]. The rather strong L3 entrapment is also illustrated in Figure 6C1 (60 min) and Figure 6C2 (180 min). We also observed the formation of “clasp”-like NET structures sticking mainly to the anterior part of the larvae (Figure 6C2, white arrow) and here we provide as supplementary data bright field- as well as fluorescence microscopy-based videos on L3 stages being entrapped by DNA-positive *agg*NET structures (Video S1 in Supplementary Material). These observations may also be linked to the induction of different types of NET by microfilarial and L3 stages. While microfilariae only induced spread and diffuse NETs, L3 additionally triggered the formation of the most robust NET type, i.e., aggNETs. Especially the latter type of NETs, which consists of rigid clusters of NET-like structures of >20 µm in diameter, may function superior to the other NET types in case of parasite entrapment of large parasite stages.

As expected, DNase I treatment significantly abolished microfilariae entrapment (*p* ≤ 0.001, Figure 6D) and minimized PMN attachment proving that NETs promoted these reactions. In contrast to NET formation, larval entrapment depended on parasite motility since dead (heat-treated) stages were entrapped to a less degree than vital ones (52 vs. 38%, respectively; *p* ≤ 0.05, Figure 6D). As also reported for NET induction, DPI treatments failed to influence parasite entrapment and led to insignificant differences when compared with untreated controls (*p* = 0.104).

To elucidate whether *D. immitis* microfilariae or L3 were killed by NETs, parasite survival was monitored over 72 h by microscopic observation of larvae motility. Overall, neither *D. immitis* microfilariae nor L3 were adversely affected by NETs within this time frame, since no differences in parasite movements were observed in comparison to non-exposed stages (please refer to L3 exposed to PMN after 72 h in Video S3 in Supplementary Material). Nevertheless, since strong NET entrapment of microfilariae was observed (see also Video S2 in the Supplementary Material), it can be speculated that in the *in vivo* scenario, either the infectivity of *D. immitis* larvae from mosquitoes might be affected due do dampened migratory capacity of these stages or the immune cell-mediated larval attack within the definitive host might be facilitated *via* this “presentation.”

## Discussion

In contrast to the vast majority of parasitic nematodes, *D. immitis* stages parasitize within the right heart and blood vessels and are thereby permanently exposed to an adverse environment, mainly composed of cells of the innate and adaptive immune system (e.g., PMN, monocytes, T cells, and NK cells), complement factors, antibodies, and cytokines/chemokines, or other soluble factors. However, investigations on early canine innate immune reactions against this parasite have scarcely been performed. Therefore, we here analyzed parasite-induced NETosis as early effector mechanism of PMN. Besides microfilariae we also included *D. immitis* L3 since this stage is transmitted to the dog as infective stage. Overall, we here provide first evidence on *D. immitis*-triggered NET release as part of early innate immune responses of canine PMN directed against microfilarial and L3 stages.

NETs are mainly formed of decondensed chromatin along with nuclear histones (H1, H2A/H2B, H3, and H4) and enzymatic granular components, such as neutrophil elastase (NE), MPO, lactoferrin, cathepsin, pentraxin, LL37, and gelatinase among others (25, 54, 55). Thus, typical NET characteristics were here confirmed for *D. immitis*-induced NETs by co-localization experiments on microfilariae and L3-triggered release of extracellular DNA being adorned with histones, NE and MPO. In accordance to studies on other metazoan parasites, L3 and microfilaria-triggered NETosis revealed as time dependent (47–50, 56). In line with data on *B. malayi, Haemonchus contortus, Strongyloides stercoralis*, and other metastrongyloid species (47–50), NETs were dissolved *via* DNase I treatment thereby proving the DNA nature of these structures. Moreover, we here observed that heat-inactivated microfilariae and L3 triggered NETs at a comparable level as vital ones, indicating that the parasites viability or integrity is not a crucial factor for NET induction. In contrast to *B. malayi* microfilariae (50), *D. immitis* microfilariae- and L3-induced NETosis appeared to be NOX-independent since DPI treatments failed to significantly inhibit NET formation.

Overall, it is noteworthy that filarial blood microfilariae appear to induce NETs independent of their ensheathment status. The microfilarial sheath is generally regarded as parasite-derived tool for improved immune evasion and was described to contain sheath-specific antigens (57, 58). The fact that both, ensheathed [*B. malayi* (50)] and non-ensheathed (*D. immitis*, this study) blood microfilariae significantly induce NET formation raises the question on surface-derived triggering molecules of these stages. However, no parasite-derived NET-inducing factors have been identified from filarial parasites, so far. Considering the fact, that microfilariae are present in blood vessels and trigger NET formation, it also appears of interest, that *D. immitis*-induced NETs may also exhibit adverse effects on the surrounding endothelium. In this context, NET-induced endothelial dysfunction and endothelial cell death was recently reported (59–61). To date, it remains unclear, whether microfilariae-triggered NET formation may also contribute to *D. immitis*-related pathogenesis with respect to vascular damage, progressive arteritis and coagulopathies, which, so far, were mainly attributed to adult stages of in chronic canine heartworm disease.

In contrast to previous NET-related reports on bacteria and selected parasites (25, 39) but in accordance to data on *B. malayi, S. stercoralis*, or *H. contortus* (47, 48, 50), *D. immitis*-triggered NETs did not promote the killing of parasite stages. As such, even after a prolonged incubation of 72 h no NET-mediated lethal effects were observed thereby rather suggesting an immobilization effect on larval stages as key mechanism of *D. immitis*-induced NETosis. So far, most studies on metazoan parasite-triggered NETs highlight the strength and efficiency of entrapment of motile large-sized pathogens when compared with bacteria, virus, fungi or protozoa. Respective features were described for *S. stercoralis, H. contortus, Angiostrongylus vasorum, Aelurostrongylus abstrusus, Troglostrongylus brevior*, and *B. malayi in vitro* as well as *in vivo* (47–50) indicating that PMN are able to recognize motile and large-sized pathogens and explicitly use NET formation as specific effector mechanism against such pathogens, as recently reported (62).

NET-mediated immobilization of *D. immitis* microfilarial stages may indeed influence the outcome of this infection since the presence of microfilariae—besides adult stages—is directly related to the pathogenesis of the disease. Thus, heartworm infections in dogs are often accompanied by pathologic alterations driven by antigen–antibody complexes (e.g., glomerulonephritis) that rely on the long-lasting circulation of microfilariae in the blood stream (17). Interestingly, circulating microfilariae drive the initiation of innate immune reactions, e.g., by a CXCR2/IL-17-dependent PMN recruitment (63). In general, the immunopathology of filarial diseases in humans and domestic animals is complex and clinical manifestations depend on the type of host immune response mounted against filarial nematodes (64). Human dirofilariosis include pulmonary granulomatous reactions especially in immune-deficient patients (9).

Interestingly, many filarial species harbor obligate bacterial endosymbionts (*Wolbachia*) which were recently shown to trigger NETosis (65). So far, it remains unclear whether microfilariae-triggered NETosis may interfere with antifilarial treatments. Efficient treatment of *D. immitis* infections in domestic dogs is currently based on macrocyclic lactones for the removal of circulating microfilariae from the blood system in addition to adulticide treatments (66). However, since evidence of resistance to this drug class was already reported in the USA (67, 68), efficacious prevention of canine dirofilariosis might be endangered in future. In this context, a strong adherence of PMN to *B. malayi* microfilariae in the presence of low ivermectin concentration and enhanced microfilarial killing by PMN or peripheral blood mononuclear cells by ivermectin treatments has been reported (69–71). Interestingly, receptor sites for macrocyclic lactones are exclusively located proximate of the excretory–secretory (ES) apparatus, which is the main site of microfilarial protein release (72). ES proteins are well recognized for their immunomodulatory properties (73) allowing parasites to evade the host immune system. Given that macrocyclic lactone administration appears to hamper ES protein release from microfilariae (71) and since attached NETs may also dampen ES release by mechanical issues, both mechanisms may contribute to an improved host systemic immune response (71). In addition, we here postulate that NET release might also facilitate larval killing by other immunocompetent cells being recruited to the site of NET formation. Previous data show that, besides PMN, other innate immune cells such as macrophages and monocytes are involved in the innate immune responses against nematode parasites. It is well known that filarial parasites induce eosinophilia and eosinophil blood count is commonly used as a screening tool (74). *In vitro* studies have demonstrated the ability of IFN-γ-activated macrophages to kill *B. malayi* microfilariae (75). Furthermore, human PMN also promote lethal effects on *B. malayi* microfilariae in the presence of autologous serum *in vitro* and extrude NETs in response to these ensheathed microfilariae thereby entangling these stages (50).

A striking feature of this study was the fact that different types of canine NETs, i.e., *diff*NETs, *spr*NETs, and *agg*NETs were observed upon contact to *D. immitis*, all of them promoting larval entrapment. Consistently, a similar phenomenon was also observed for *H. contortus*- and lungworm-induced NETs (48, 49) showing a tight immobilization of L3 by these NET structures. However, concerning L3-induced *agg*NETs, it may also be speculated that they function in the prevention of proper L3 exsheathment into L4 stages within the definitive host and thereby hamper the ongoing development of *D. immitis*.

The here reported types of *D. immitis*-mediated NETs might be linked to the recently described capacity of mammalian PMN to sense small and large-sized pathogens and to selectively release NETs in response to large pathogens Branzk et al. (62). Given that especially L3 stages, but also microfilariae of *D. immitis*, represent large-sized pathogens, it appears obvious that PMN rather react by NETosis than by phagocytosis which will be ineffective against large multicellular pathogens.

Overall, we here present new insights into the early host innate immune response driven against the zoonotic parasite *D. immitis*. We demonstrate for the first time that both, microfilariae and L3 are potent inducers of different types of canine NETs. Overall, canine NETs do not kill the parasitic stages but might facilitate their killing by other leukocytes circulating in the blood system as postulated elsewhere. So far, it remains unclear whether excessive NET formation may also have adverse effects for the definitive host, such as vascular damage or coagulopathies. Consequently, we here call for more investigations not only on the *in vivo* evidence of *D. immitis*-triggered NETosis but also on its intravascular consequences with regards on pathogenesis and outcome of disease.

## Ethics Statement

This study was carried out in accordance with the recommendations of the local animal care and use committee and by governmental authorities (LANUV #200/A176 and #200/A154).

## Author Contributions

CH, AT, DK, TMC, and IC designed the project and experiments. DK and AP isolated, purified, and provided the parasites as well as the blood for this study. TMC, IC, EZ, and UG carried out most of the experiments. TMC, AT, CH, and IC prepared the manuscript. All the authors reviewed the manuscript.

## Conflict of Interest Statement

The authors DK and AP are employed at Bayer Animal Health GmbH, Leverkusen, Germany. The authors declare that this study was mainly financed by the Institute of Parasitology, Justus Liebig University Giessen, Germany and partially supported by Bayer Animal Health GmbH, Leverkusen, Germany. The authors DK and AP (employed at Bayer Animal Health GmbH, Leverkusen, Germany) participated in design of project and experiments by isolating, purifying and providing parasites and blood for the current investigation and reviewed manuscript. All other authors declare no competing interests.

## Supplementary Material

Video S1 *D. immitis* L3 entrapped in NETs structures. Video performed using fluorescence microscopy analysis along with the DNA-marker Sytox Orange to stain NETs-DNA contacting *D. immitis* L3.

Video S2 *D. immitis* microfilariae entrapped in NETs. By using bright field microscopy, *D. immitis* microfilariae is observed entrapped in NETs.

Video S3 *D. immitis* L3 are not killed by NETs after 72 h exposure to canine PMN. By using bright field microscopy, *D. immitis* L3 is observed still motile entrapped in *agg*NETs after 72 h of coculture with canine PMN.

Image S1 **(A,B)** *D. immitis*-induced NETosis after 10 min of incubation with canine PMN. Merge images on bright field and fluorescence microscopy obtained by using DNA-staining Sytox Orange and an anti-histone antibody.

Image S2 *D. immitis* microfilariae induce *agg*NETs and *diff*NETs. As indicated by arrows, the formation of different types of NETs is demonstrated as seen in merge image using bright field and fluorescence microscopy analysis with DNA-staining Sytox Orange and an anti-histone antibody.

Image S3 *D. immitis* microfilariae induce *spr*NETs. As indicated by arrows, the formation of different types of NETs is demonstrated as seen in merge image using bright field and fluorescence microscopy analysis with DNA-staining Sytox Orange and an anti-histone antibody.
