# Supplementary figures and images for: Dirofilaria immitis Microfilariae and Third-Stage Larvae Induce Canine NETosis Resulting in Different Types of Neutrophil Extracellular Traps
Source: Front Immunol. 2018 May 8;9:968. doi: 10.3389/fimmu.2018.00968 (PMC5951940; doi:10.3389/fimmu.2018.00968)

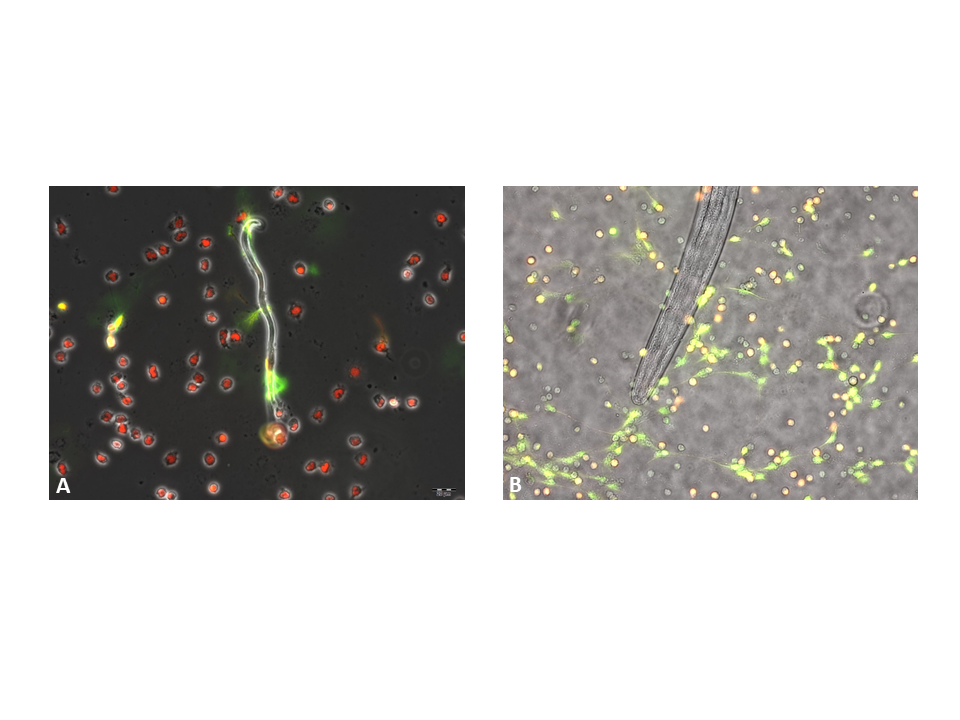

Supplement: Image S1 — (A,B) D. immitis-induced NETosis after 10 min of incubation with canine PMN. Merge images on bright field and fluorescence microscopy obtained by using DNA-staining Sytox Orange and an anti-histone antibody. [file Image_1.TIF]

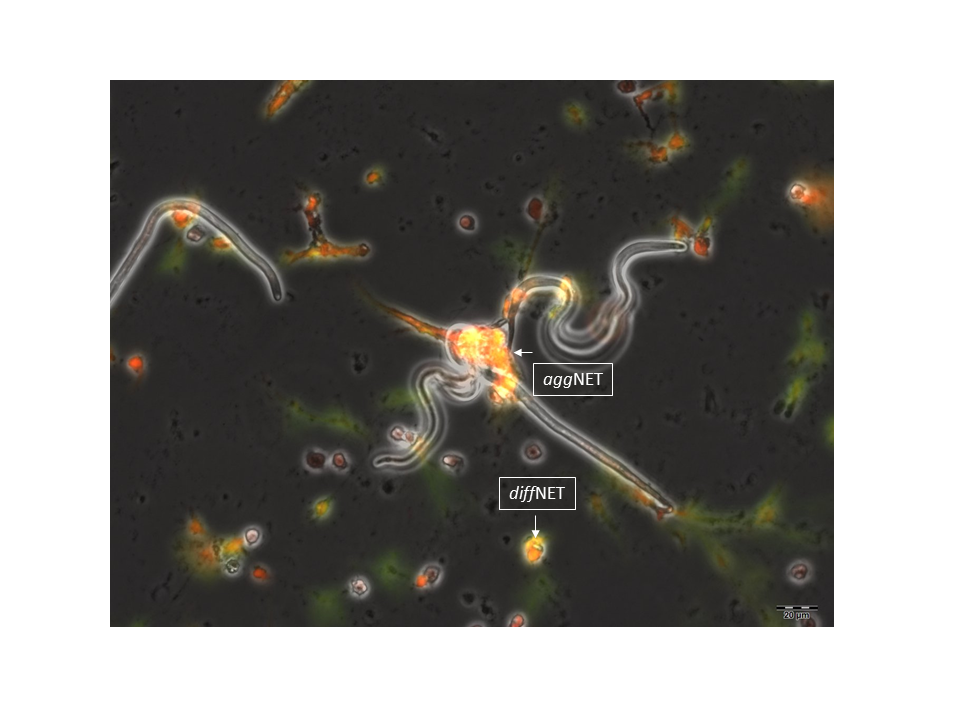

Supplement: Image S2 — D. immitis microfilariae induce aggNETs and diffNETs. As indicated by arrows, the formation of different types of NETs is demonstrated as seen in merge image using bright field and fluorescence microscopy analysis with DNA-staining Sytox Orange and an anti-histone antibody. [file Image_2.TIF]

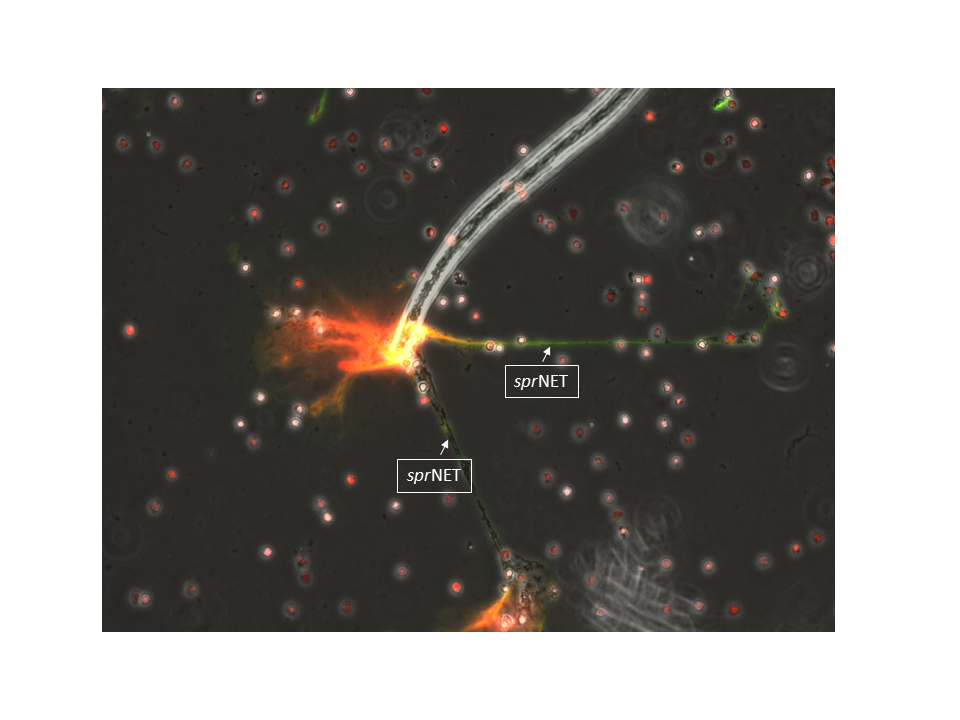

Supplement: Image S3 — D. immitis microfilariae induce sprNETs. As indicated by arrows, the formation of different types of NETs is demonstrated as seen in merge image using bright field and fluorescence microscopy analysis with DNA-staining Sytox Orange and an anti-histone antibody. [file Image_3.TIF]
